# Cerebral Small Vessel Disease: Therapeutic Approaches Targeting Neuroinflammation, Oxidative Stress, and Endothelial Dysfunction

**DOI:** 10.3390/cimb47040232

**Published:** 2025-03-27

**Authors:** Habibe Yılmaz, Ulvi Bayraktutan

**Affiliations:** 1 Academic Unit of Mental Health and Clinical Neurosciences, University of Nottingham, Nottingham NG7 2UH, UK; 2 Department of Pharmaceutical Biotechnology, Faculty of Pharmacy, Trakya University, Edirne 22030, Türkiye

**Keywords:** brain, small vessel disease, anti-anginal drugs, acetylcholine esterase inhibitors, HMG-CoA reductase inhibitors, lithium drugs, phosphodiesterase inhibitors, oral antihyperglycaemic drugs, tetracycline antibiotics

## Abstract

Cerebral small vessel disease (cSVD) is a common cause of stroke and dementia. Ageing, hypertension, hyperglycaemia, and smoking make up the biggest risk factors for cSVD. They individually or collectively increase the levels of reactive oxygen species, pro-inflammatory cytokines and matrix metalloproteinases, decrease the bioavailability of nitric oxide, and, in the process, compromise the structural integrity and function of the vascular endothelium, blood–brain barrier, and brain parenchyma. These then appear as white matter hyperintensities, enlarged perivascular spaces, cerebral microbleeds, and atrophy in cerebral imaging. As there is currently no curative therapy for cSVD, prevention or delay of cSVD remains of particular importance to preserve quality of life for as long as possible. Bearing that in mind, this review explores whether drugs used for other neurovascular conditions may prevent neuroinflammation and oxidative damage and effectively maintain endothelial function and blood–brain barrier integrity. It also examines whether potential benefits may be extended to cSVD. The list of drugs includes anti-anginal drugs, acetylcholine esterase inhibitors, β-hydroxy β-methylglutaryl-CoA reductase inhibitors, lithium drugs, phosphodiesterase inhibitors, oral antihyperglycaemic drugs, and tetracycline antibiotics. This review discusses the mechanisms of action of these agents and critically evaluates preclinical, translational, and clinical research pertaining to cSVD.

## 1. Introduction

Using the available data from 2021, the World Health Organisation (WHO) has recently released the list of the top 10 causes of death in 2024. While ischaemic heart disease continued to be the leading cause of death, stroke came third in the list after COVID-19. Alzheimer’s disease (AD) and other dementias also ranked amongst the leading causes of death [1]. The statistics from the WHO indicate that each year, globally, approximately 15 million people suffer a stroke. While one-third of these patients recover from it, another one-third are left permanently disabled and the remaining one-third sadly die from stroke [2]. According to data from the Stroke Association, stroke is also one of the leading causes of death in the UK [3]. The future projections concerning the stroke incidence and prevalence in the UK predict about 60% and 120% increases by 2035, respectively [4].

Stroke is characterised by pathological alterations resulting from disrupted cerebral blood flow and divided into two main types: haemorrhagic or ischaemic [5]. Cerebrovascular haemorrhages occur when an artery within or on the surface of the brain is ruptured. One this happens, the blood is extravasated into the brain parenchyma, ventricles, or the subarachnoid space. This may be precipitated by elevated blood pressure, the use of anticoagulants or antithrombotic agents, or head trauma [6]. Ischaemic strokes are, on the other hand, characterised by an obstruction in the vessels leading to or in the brain due to thrombus or embolism. Ischaemic strokes account for about 85% of all stroke cases in Western populations [7]. Large artery atherosclerosis, cardiac embolism, and cerebral small vessel disease (cSVD) represent the most common causes of ischaemic strokes. cSVD arises from a variety of pathological alterations that impair the cerebrovascular network, including the cerebral arteries, capillaries, arterioles, and venules [8]. It accounts for ~25% of all ischaemic stroke cases and about 45% of all dementia cases. The incidence of this condition escalates with advancing age, irrespective of differences in race or sex [9]. Hypertension, smoking, diabetes mellitus (DM), obstructive sleep apnoea, chronic kidney disease, and branch atheromatous disease are amongst the main risk factors involved in the pathogenesis of cSVD [10].

There is currently no cure for cSVD and available therapeutic strategies focus on addressing associated risk factors and promoting lifestyle modifications. Hence, exploration of the potential pharmaceutical interventions that could modify the progression of cSVD has been at the forefront of recent research. In this regard, scavengers of reactive oxygen species (ROS), drugs that enhance endothelial nitric oxide synthase (eNOS) activity and restore normal endothelial function, matrix metalloproteinase inhibitors, and agents that strengthen the integrity of tight junctions (TJs) and the blood–brain barrier (BBB) have attracted some attention [11,12,13]. Similarly, cytokine inhibitors and microglial modulators that aim to regulate neuroinflammation, calcium channel blockers that modulate cerebral blood flow, and cyclic guanosine monophosphate (cGMP) enhancers have also been under investigation [14,15,16,17,18].

All relevant studies from all years, including both reviews and original articles, were identified in the PubMed database by a literature search using the key MeSH terms “cerebral small vessel disease”, “ischaemic stroke”, “tetracycline antibiotics”, “anti anginal drugs”, “acetylcholine esterase inhibitors”, “HMG-CoA reductase inhibitors”, “lithium drugs”, “phosphodiesterase inhibitors”, “in vitro blood-brain barrier models”, and “oral antihyperglycaemic drugs”. A Nottingham University search, ClinicalTrials.Gov, and Google Scholar were also used to collect pertinent studies.

## 2. Cerebral Small Vessel Disease

Cerebral SVD is a cerebrovascular syndrome characterised by the progressive deterioration of small penetrating arteries, arterioles, capillaries, and venules. It encompasses a broad spectrum of conditions including endothelial dysfunction, BBB disruption, neuroinflammation, oxidative stress, and impaired cerebral autoregulation. These lead to white matter hyperintensities (WMHs), lacunar infarctions, microhaemorrhages, and cerebral atrophy and, as a consequence, trigger stroke, vascular dementia, and accompanying cognitive impairments [19,20].

cSVD markers can be categorised as imaging, non-genetic biomarkers from fluids, and genetic markers. Magnetic resonance imaging (MRI) is considered the gold standard for imaging-based diagnosis. WMHs can be detected via T2-weighted MRI. Quantitative and volumetric analyses of these lesions provide valuable insights into the progression and severity of the underlying cSVD [21,22]. MRI commonly shows small, cavitated lesions known as lacunes, resulting from damage to deep penetrating arteries. The presence of lacunes is associated with a high risk of stroke and dementia [23]. Cerebral microhaemorrhages (CMHs) may be detected using susceptibility-weighted imaging. CMHs indicate microvascular damage and haemorrhage-prone vessels, mostly in amyloid-associated angiopathies and hypertensive cases [24]. Enlarged perivascular spaces (EPSs), which signify microvascular impairment, impaired glymphatic clearance, and neuroinflammation, may also be present [25].

Non-genetic biomarkers of fluids include neurofilament light chain (NfL), amyloid-β and tau proteins, and inflammatory endothelial dysfunction markers. Increased levels of NfL in the cerebrospinal fluid (CSF) and blood are indicative of axonal damage and neurodegeneration [26]. Elevated concentrations of amyloid-β and tau proteins in the brain correlate with Alzheimer’s disease and indicate vascular impairment [27]. Increased plasma levels of interleukin-6 (IL-6), C-reactive protein (CRP), and tumour necrosis factor-α (TNF-α) pinpoint systemic inflammation and contribute to endothelial dysfunction in cSVD [28,29,30]. Similarly, circulating vascular endothelial growth factor (VEGF) and soluble intercellular adhesion molecule-1 (SICAM-1) also indicate the presence of endothelial dysfunction [31].

Cerebral autosomal dominant arteriopathy with subcortical infarcts and leukoencephalopathy (CADASIL) is the most common genetic risk factor for cSVD. Genetic mutations in the neurogenic locus notch homolog protein 3 (NOTCH3) gene, which produces a transmembrane protein found in vascular smooth muscle cells, lead to the formation of misfolded NOTCH3 protein and granular osmiophilic material. This in turn results in impaired vascular function and alterations to white matter [32]. Cerebral autosomal recessive arteriopathy with subcortical infarcts and leukoencephalopathy (CARASIL) is associated with the HtrA serine peptidase 1 (HTRA1) gene mutation, which encodes a serine protease. Transforming growth factor-β (TGF-β) accumulation is observed in individuals with HTRA1 gene mutations, leading to vascular fibrosis and progressive ischaemic injury [33]. Retinal vasculopathy with cerebral leukodystrophy and systemic manifestations (RVCL-S) is caused by mutations in the three prime repair exonuclease 1 (TREX1) gene, which encodes a DNA exonuclease. Consequently, there is an accumulation of cytosolic deoxyribonucleic acid (DNA) and subsequent activation of the innate immune system, which leads to vascular inflammation and endothelial dysfunction [34]. Collagen α-1(IV) chain (COL4A1) and collagen α-2(IV) chain (COL4A2) genes encode type IV collagen. Genetic alterations in this region disrupt the integrity of the basal membrane. The vessels become susceptible to haemorrhage and ischaemia, leading to recurrent strokes, white matter lesions, and CMBs [35]. Fabry disease is a genetically inherited disorder linked to the X chromosome and arises from pathogenic variants in the galactosidase α (GLA) gene. The accumulation of globotriaosylceramide within endothelial cells can lead to the occlusion of small vessels, alterations in white matter, and the occurrence of stroke [36]. Familial idiopathic basal ganglia calcification, known as Fahr’s disease, is caused by mutations in the platelet-derived growth factor receptor-β (PDGFRB) gene. As a result of this mutation, pericyte function is impaired and the BBB integrity is disrupted [37].

**Figure 1:**
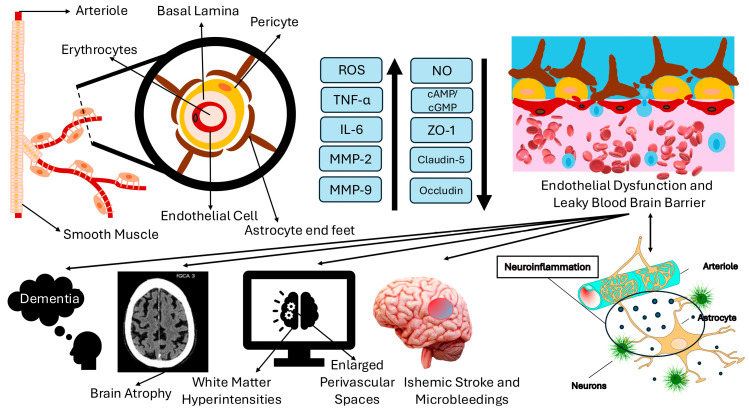
The blood–brain barrier consists of endothelial cells, pericytes, astrocytic end feet, and the basal lamina. Increased availability or activity of ROS, pro-inflammatory cytokines such as TNF-α and IL-6, and MMPs along with decreases in NO, cAMP/cGMP, and tight junction proteins ZO-1, claudin-5, and occludin levels cause endothelial dysfunction. This in turn increases the permeability of the BBB, allows the passage of inflammatory compounds to brain parenchyma, and exacerbates BBB damage. As a result, ischemic stroke, cerebral microhaemorrhages, WMH, and EPSs are observed. Unless medically intervened with, brain atrophy and dementia will emerge. ROS: Reactive oxygen species, TNF-α: Tumour necrosis factor α, IL-6: Interleukin-6, MMP: Matrix metalloproteinase, NO: Nitric oxide, cAMP: Cyclic adenosine monophosphate, cGMP: Cyclic guanosine monophosphate, ZO-1: Zonula occludens-1.

Impaired function of the endothelium, including disturbances in the small penetrating arteries, can result in reduced cerebral perfusion and ischaemia, which are associated with white matter abnormalities [19,38,39]. Endothelial dysfunction emerges from and leads to reduced bioavailability of NO, which impairs vasodilatation and promotes vascular inflammation. The increased vascular permeability and disruption of the BBB further contribute to inflammation and oedema and correlate with CMHs [11,38,39]. Vascular smooth muscle dysfunction, on the other hand, is linked to the severity of cSVD [39]. The reduced bioavailability of NO, derived in part from the downregulation of eNOS, elicits a marked elevation in superoxide anion levels and other ROS [40]. It is of note that the diminished expression of claudin-5 and occludin proteins within the TJs, which regulate the selective permeability of substances, also disrupts the function and integrity of the BBB and thus facilitates the infiltration of inflammatory mediators into the brain parenchyma. Degradation of the extracellular matrix and myelin-supporting proteins by enhanced activities of matrix metalloproteinases (MMPs), in particular MMP-2 and MMP-9, further exacerbate BBB and brain tissue disruption following the onset of endothelial dysfunction. Heat shock protein 90α (HSP90α), synthesised by dysfunctional endothelial cells (ECs), also prevents oligodendrocyte maturation, causing myelin loss and white matter changes. Taken together, all these factors contribute to BBB disruption, inflammatory cell infiltration, and the observation of the so-called WMHs on MRI [41,42,43]. Figure 1 summarises the information discussed in this section.

## 3. BBB Integrity in Cerebral Small Vessel Disease and In Vitro Models of the BBB

The BBB is one of the three barriers in the central nervous system (CNS). ECs, pericytes, and astrocytes make up the main cellular components of the BBB. The ECs in the CNS cover the entire inner surface of all cerebral blood vessels and, in the process, form a continuous and complete basement membrane without pores. As well as being a physical barrier, the BBB is also a biochemical barrier [44]. The continuous tight junctions, formed by proteins like claudins and occludin, supported by accessory proteins like zonula occludens-1 (ZO-1), between capillary ECs, the basement membrane, and the extracellular matrix; perivascular neurons; pericytes; and astrocytic foot processes maintain the normal BBB function and limit the entry of potentially harmful chemicals into the CNS [45,46]. While ECs form a highly selective permeable barrier, astrocytes contribute to BBB integrity by regulating ion homeostasis, vascular function, and neuroimmune responses. Pericytes communicate with ECs to regulate TJ formation, vascular stability, and neuroinflammation. As pericytes are embedded in the EC lining, any pathology affecting their numbers and function can also affect the integrity of the BBB [46,47].

As alluded to above, BBB hyperpermeability constitutes an important part of brain damage. It is also regarded as an early pathology of cSVD in that decreased expression and/or mislocalisation of key TJ proteins occludin and claudin-5 due to oxidative stress, inflammation, and vascular damage play a key role [48]. Breakdown of the BBB facilitates the infiltration of plasma proteins, immune cells, and pro-inflammatory cytokines to the brain parenchyma, which activate microglia and astrocytes and promote the generation of more cytokines and ROS. In addition to the BBB, neurovascular coupling (NVC) is also disrupted during cSVD. This culminates in chronic hypoperfusion, which exacerbates oxidative stress, damages ECs, and inflicts damage on the white matter [49]. Oxidative stress arises when the balance between ROS production and the body’s antioxidant defence is disrupted. Elevated ROS levels target polyunsaturated fatty acids in endothelial cell membranes, triggering lipid peroxidation and the release of reactive aldehydes such as malondialdehyde and 4-hydroxynonenal, which, in turn, bind to DNA and cause strand breaks. Through oxidizing cysteine residues and disrupting disulphide bonds in tight junction proteins like ZO-1, occludin, and claudin-5, ROS destabilize them. Additionally, ROS can activate MMPs and further compromise the structural integrity of these proteins. As a consequence, the breakdown of tight junctions creates gaps between neighbouring endothelial cells and promotes BBB permeability. As the barrier weakens, harmful proteins such as fibrinogen, thrombin, and albumin, along with immune cells, infiltrate the brain. The cytokines released from these immune cells, including TNF-α and IL-6, activate the NF-κB and MAPK pathways, amplifying pro-inflammatory cytokine production. This escalation in neuroinflammation accelerates tissue damage and exacerbates disease progression [50,51].

In cSVD, the extent of changes in BBB disruption correlates positively with the magnitude of the changes in WMH and CMH on MRI. It is noteworthy here that the level of BBB disruption varies from one region to another in different cSVD types [52,53]. Taken together, these findings indicate that therapeutic strategies aimed at preserving the integrity and function of the BBB are more efficacious in reducing the burden of disease compared to a symptomatic approach.

**Figure 2:**
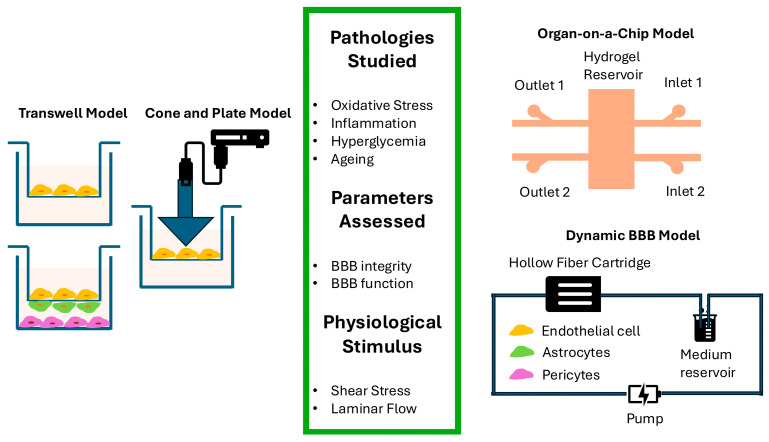
In vitro BBB models used to investigate the impact of pathologies associated with cSVD on the blood–brain barrier. All models illustrated in the figure can effectively assess the impact of different pathologies on barrier integrity and function. The cone and plate, BBB-on-a-chip, and dynamic models allow the assessment of these pathologies in the presence of key physiological stimuli, notably shear stress and blood flow.

The use of in vitro models of the BBB in cerebrovascular research is highly informative. The models have evolved greatly in recent years and now include both dynamic and static models. The static two-dimensional (2D) models represent the most accessible, cost-effective, and widely used models. Triple cell culture models, enabling intercellular interactions amongst ECs, astrocytes, and pericytes on transwell inserts, remain the most commonly used models of the human BBB in laboratory conditions [54,55,56]. Through incorporation of neurons into the system, these models have since become a quadruple cell system [57]. In recent years, static three-dimensional (3D) models using horizontal microfluidics support, organoid models, and models utilising bioprinted scaffolds to mimic the lumen structure have also become available. For dynamic models, single- or multi-channel vertical microfluidic systems have been used to generate both 2D and 3D models [58,59]. To simulate shear stress as a dynamic effect, the cone and plate model, which can be adapted to transwell systems, has emerged [60]. More sophisticated dynamic in vitro BBB (DIV-BBB) systems using hollow-fibre cartridges, a flow path, and a reservoir have also been developed [61]. In recent years, viable perfusion-based systems have been explored as an alternative to the use of hollow fibres for the preparation of the DIV-BBB [62]. Figure 2 illustrates the commonly used in vitro models of the human BBB.

Static 2D BBB models remain among the most effective tools for investigating the molecular mechanisms underlying cSVD. They can specifically assess the impact of various cSVD-related pathologies such as hyperglycaemia, oxidative stress, inflammation, and ageing on barrier integrity and function in the absence or presence of various so-called therapeutics. Recent studies using triple cell culture models have attributed hyperglycaemia-mediated impairments in BBB integrity to tight junction protein dissolution, stress fibre formation, and increased release of endothelin-1, IL-8, and basic fibroblast growth factor [45]. Using the same model, another study has coupled the TNF-α-induced BBB dysfunction to the upregulation of endothelial Rho kinase expression [29]. Previous studies have reported that TNF-α also increases MMP-2 activity, actin stress fibre formation, caspase activity, NADPH oxidase activity, and O_2_^−^ production in both HBMEC and HA [63]. Oxidative stress emerging from ischemia-reperfusion injury, mimicked experimentally by oxygen–glucose deprivation alone or followed by reperfusion (OGD ± R), has also appeared to compromise cerebral barrier integrity through increases in MMP-2/9 and NADPH oxidase activity, TNF-α and O_2_^−^ secretion, apoptosis, and actin stress fibre formation [64]. These findings were supported by the findings of another study subjecting 2D or 3D BBB models to different concentrations of H_2_O_2_ to investigate the effect of acute and chronic oxidative stress [65]. Finally, BBB models set up with stress-induced or replicative senescent endothelial cells have also corroborated the barrier-disruptive effects of ageing on BBB characteristics, where alterations in the expression and subcellular localisation of tight junction proteins ZO-1 and claudin-5 and MMP-2 activity appeared to be instrumental [66,67,68]. Organ-on-a-chip models enable the simulation of shear stress and fluctuating blood flow, key factors linked to hypertension—a major risk factor for cSVD—while also allowing the examination of molecular-level changes observed in cSVD pathology [69,70].

The literature concerning the barrier-restorative effects of the outlined specific pharmacological agents within the context of cSVD or associated risk factors is extremely scant. However, there are studies highlighting the molecular mechanisms involved in their barrier-protective effects. For instance, oral antidiabetic metformin (10 mM) has been shown to attenuate BBB disruption in rodents after middle cerebral artery occlusion [71], which may be explained by its ability to activate AMP-activated protein kinase in endothelial cells in a dose-dependent manner [72]. Similarly, lithium carbonate (1.25–10 mM) has also been shown to alleviate BBB breakdown after cerebral ischaemic injury in mice by upregulating Wnt/β-catenin signalling [73] and MAPK/ERK1/2 activity [74]. The effects of atorvastatin also appear to be dose-dependent, where higher doses (0.2 to 25 μM) evoke endothelial dysfunction via the disruption of mitochondrial respiration in endothelial cells [75] and BBB failure at a dose range of 45 to 90 μM [76]. Interestingly, the doses applied in these studies were higher than the pharmacological dose ranges for metformin (20–30 µM), lithium (0.4–1.2 mM), and atorvastatin (4–15 nM) [77,78,79]. However, the effective cerebrovascular protective dose for minocycline HCl (400 nM) following OGD-R injury [80] was considerably lower than the recommended serum concentration of 6.1 to 17.7 mM [81]. It is noteworthy here that in cell culture conditions and animal experiments, the doses of most pharmaceutical agents tend to be higher than their corresponding plasma or serum concentrations in humans. This is in part because only minute amounts of the drug remain in the circulation when a drug is highly tissue-bound. In other words, a low plasma concentration indicates a high volume of distribution and vice versa [82]. Secondly, in most cases, in vitro concentrations of drugs have to be markedly higher than the corresponding in vivo plasma concentration in order to produce similar biological effects in the target cells [83]. Finally, the rate of drug elimination is faster in small laboratory animals than humans, a well-documented difference in pharmacokinetics [84].

The BBB models developed over time have aimed to better reflect human physiology or pathophysiology. However, there is a standardisation problem for dynamic and sophisticated models in drug research. As sophisticated models demand considerable expertise and time to establish, they are not widely used in laboratory studies. However, selecting an appropriate model is crucial for the research question to be addressed. For instance, triple culture systems are widely used for screening drugs and investigating the molecular mechanisms involved in neurovascular pathologies [56]. The use of human primary cells in these models increases the translatability of the results to clinical settings [85]. Although in vitro models are not sufficient to exactly mimic in vivo conditions, it is anticipated that for studies like cSVD, where the pathology and molecular mechanisms are not yet fully understood, the use of cell culture models of the human BBB will be highly beneficial.

## 4. Current Pharmacological Interventions for cSVD

Considering the pathologies and molecular pathways involved in cSVD, some medicines are likely to be effective in its management. The mechanisms of action for each drug group and their relationship with the well-documented causes of cSVD are summarized below to provide the reader with an overview.

### 4.1. Acetylcholine Esterase Inhibitors

Acetylcholine (ACh), an essential excitatory neurotransmitter synthesised by cholinergic neurons, is closely linked to learning, memory, and various behaviours. ACh deficiency is therefore closely associated with the pathogenesis of Alzheimer’s disease, a form of dementia [86]. ACh is found in nerves, muscles, and the central and peripheral nervous systems. In the peripheral nervous system, it operates at the connections between motor nerves and skeletal muscle. In the CNS, it has been identified primarily between neurons and in several important long-axon cholinergic pathways. Within the cholinergic system, acetylcholinesterase hydrolyses ACh to choline. The released choline is then reuptaken into the presynaptic terminal via the high-affinity choline uptake process. It is subsequently converted back to ACh by the choline acetyltransferase enzyme using choline and acetyl coenzyme A. In the presence of acetylcholinesterase (AChE) inhibitors, the degradation of ACh is prevented and its accumulation in the synaptic cleft is ensured, thus keeping postsynaptic receptors activated and prolonging cholinergic receptor signalling [87].

In cerebral ischaemia, cholinergic system dysfunction and post-ischaemic inflammation have crucial effects on vascular dementia. It has been shown that in cases of arterial occlusion, there is a loss of cholinergic nerves, a decrease in choline acetyltransferase and AChE activities, and a reduction in ACh levels in the neocortex, hippocampus, and CSF. Similar findings have been reported in post-mortem patient studies. As a result of the inflammatory processes following cerebral ischaemia, immune cells secrete ROS and various toxic components such as pro-inflammatory cytokines, which damage neurons and disrupt the BBB integrity. BBB disruption can aggravate the inflammatory state by causing a secondary ischaemic attack. However, ACh secreted by functional cholinergic neurons can bind to the α-7 nicotinic acetylcholine receptors of nearby immune cells and suppress cytokine release in a reaction termed the “cholinergic anti-inflammatory pathway” [88]. In brief, the activation of the vagus nerve by pro-inflammatory cytokines leads to the release of ACh from efferent vagal nerve terminals. ACh then interacts with α7nAChR on macrophages, glial cells, and other non-neuronal cells, blocking the nuclear translocation of NF-κB, which induces the expression of many pro-inflammatory genes, including those encoding cytokines and chemokines. Additionally, this interaction triggers the phosphorylation of JAK2 and STAT3, promoting the movement of STAT3 into the nucleus, which then lowers the expression of various cytokines, including TNF-α, macrophage inflammatory protein 2, and IL-6 [89,90]. By inhibiting AChE, the breakdown of ACh can be prevented, potentially increasing its levels and facilitating its interaction with α7nAChR. By binding to α7nAChR, ACh may reduce the production of both local and peripheral pro-inflammatory cytokines, thereby decreasing local inflammation as well as the leakage of pro-inflammatory cytokines from the blood in conditions where the BBB integrity is compromised. Consequently, inflammation and the associated tissue damage are reduced.

NVC, defined as the tight relationship between neurons and haemodynamic responses, is crucial for brain function and is utilised in functional magnetic resonance imaging (fMRI), positron emission tomography (PET), and single-photon emission computed tomography (SPECT) brain imaging techniques, where neural activity is examined alongside vascular responses [91]. ACh has been shown to regulate cerebral blood flow in NVC. ACh exerts this effect through its vasodilatory properties, either by activating eNOS or indirectly by stimulating interneurons containing neuronal nitric oxide synthase (nNOS), which secrete NO [92]. This pathway appears to be one of the important factors in the preservation of BBB integrity in vascular dementia.

AChE inhibitors are divided into two categories: reversible and irreversible. Irreversible AChE inhibitors encompass compounds such as diazinon, diazoxon, and malathion diethylmaleate, which are organophosphorus compounds. As these irreversible inhibitors are generally associated with toxic effects, it is unlikely that they possess any therapeutic properties [93]. In contrast, reversible AChE inhibitors, such as donepezil, rivastigmine, and galantamine, improve ACh-mediated vasodilation by modulating eNOS activity and, as a consequence, enhance cerebral perfusion [94]. By increasing ACh levels, they may contribute to the activation of the cholinergic-anti-inflammatory pathway and attenuate or prevent neuroinflammation, a hallmark of cSVD. This effect is largely achieved by decreasing the release of pro-inflammatory cytokines such as TNF-α and IL-1β [95]. Moreover, ACh helps to preserve BBB integrity by reducing oxidative stress and modulating tight junctional complex formation [96].

### 4.2. β-Hydroxy β-Methylglutaryl-CoA (HMG-CoA) Reductase Inhibitors

HMG-CoA reductase inhibitors, a group of anti-hyperlipidaemic drugs also known as statins, are medications used to control the deleterious effects of lipids and overall cardiovascular disease risk. They act by competitively binding to the β-Hydroxy β-methylglutaryl-CoA reductase enzyme, which is involved in the rate-limiting step of the cholesterol synthesis pathway, thereby preventing the conversion of HMG-CoA to mevalonate. They reduce the intracellular cholesterol level and increase the expression of low-density lipoprotein (LDL) receptors on the cell surface, thus reducing plasma LDL levels [97,98]. Decreased plasma cholesterol levels activate sterol regulatory element binding protein-2 (SREBP-2), leading to upregulation of LDL receptor gene expression, which further diminishes LDL-C levels in the circulation [99]. In addition, inhibition of mevalonate synthesis reduces the synthesis of isoprenoid derivatives that are involved in post-translational modification of proteins involved in cell signalling. This accounts for the well-known pleiotropic effects of statins, including both anti-inflammatory and anti-proliferative effects [100]. Indeed, statins can reduce systemic inflammation and cytokine production by inhibiting the nuclear factor-kappa B (NF-κB) pathway. The pleiotropic benefits of statins also include a reduction in oxidative stress, improvement of endothelial function, and prevention of thrombus formation and platelet aggregation through modulation of isoprenoid intermediates [101,102]. Increased NO production by eNOS and decreased production of ROS by NADPH oxidase, a pro-oxidant enzyme, play a key role in statin-mediated improvements observed in endothelial function [103,104,105]. Due to their suppressive effects on ROS and ensuing oxidative damage, statins help maintain TJs between ECs [106,107].

### 4.3. Lithium Drugs

Lithium salts, especially lithium carbonate and lithium citrate, have been used as mood stabilisers in bipolar disorder for many years. They show these effects by regulating neural signalling and neuroplasticity through complex and multiple pathways [108]. Lithium inhibits inositol monophosphatase, the key enzyme in the phosphatidyl inositol pathway. As a result of this inhibition, it reduces the conversion of free inositol to inositol-1-phosphate, thus reducing the substrate required for phosphatidylinositol-4,5-bisphosphate synthesis. This diminishes inositol triphosphate and diacylglycerol phosphate activity, modulating calcium signalling and protein kinase C activity, which are known to elevate during early phases of ischaemic stroke due to BBB permeability [109,110]. Lithium also directly inhibits glycogen synthase kinase-3 (GSK-3), which is involved in many cellular processes such as the circadian rhythm, neurogenesis, and apoptosis. GSK-3 phosphorylation often interferes with the activity or stability of its targets, including glycogen synthase and β-catenin. Inhibiting GSK-3 enhances BBB function by stabilizing proteins that extend the half-life of tight junction proteins occludin and claudin-5. Furthermore, GSK-3 inhibition reduces the release of pro-inflammatory cytokines and adhesion molecules like ICAM-1 and VCAM-1 from endothelial cells. This also leads to a decrease in GSK-3-mediated immune-endothelial activity and leukocyte trans-endothelial migration. As indicated above, lithium enhances the activity of several transcription factors such as β-catenin and thus promotes cell survival and neuroplasticity [111,112]. Lithium also up-regulates brain-derived neurotrophic factor, which promotes hippocampal neurogenesis, neural survival, and synaptic plasticity [113,114]. By increasing oxidative phosphorylation and adenosine triphosphate production in mitochondria, lithium regulates cellular energy homoeostasis, as required for high neural activity [115,116]. In addition, it reduces ROS production by inducing superoxide dismutase activity [117,118] and improves vascular relaxation and CBF by enhancing the bioavailability of NO through a mechanism involving an increase in β-catenin levels [119]. Through mediating a decrease in occludin and claudin-5 turnover, via the inhibition of GSK-3, lithium has also been coupled with BBB integrity and overall vascular homeostasis [120].

### 4.4. Phosphodiesterase Inhibitors

Phosphodiesterase (PDE) inhibitors are used in the treatment of many diseases such as cardiovascular diseases, erectile dysfunction, pulmonary hypertension, and inflammatory disorders. PDE inhibitors selectively target each isoform of the phosphodiesterase enzyme family, which includes 11 isoforms [121]. Drugs that target the PDE3 isoform in cardiovascular tissue, such as cilostazol and milrinone, increase cyclic adenosine monophosphate (cAMP) levels, whereby they evoke protein kinase A (PKA) activity and augment vasodilatory responses [122]. The PDE4 isoform is primarily localised within immune and inflammatory cells. As anticipated, PDE4 inhibitors trigger anti-inflammatory responses by elevating cAMP levels and diminishing the production of major pro-inflammatory cytokines, including TNF-α and IL-6. PDE4 inhibitors prevent chronic inflammation in NVU and are commonly preferred therapeutics for chronic obstructive pulmonary disease and asthma [123]. The PDE5 isoform is highly expressed in vascular smooth muscle and promotes cGMP hydrolysis. It provides relaxation and vasodilation of smooth muscle by stimulating the NO-cGMP signalling pathway. It is widely used in the treatment of erectile dysfunction [124]. By increasing cGMP levels, PDE5 inhibitors also stimulate microvascular perfusion and decrease the risk of ischaemic damage [125,126]. While PDE1 and PDE2 inhibitors are believed to have beneficial effects on neural signalling and cardiac function by modulating the cAMP and cGMP pathways due to their dual-specific functions, PDE7 and PDE8 inhibitors are considered for the treatment of autoimmune diseases and neuroinflammatory conditions because they selectively regulate cAMP levels in immune cells [127,128]. PDE inhibitors that are effective on PDE3, PDE4, and PDE5 also support neurogenesis and synaptic plasticity by modulating the cAMP-responsive element-binding protein (CREB) pathway [129].

Data pertaining to the effects of PDE inhibitors on WMHs remain inconclusive. Indeed, while treatment with ibudilast, a PDE4 inhibitor, has been reported to reduce lesions in rats subjected to chronic hypoperfusion by about 50% and 70% at doses of 30 mg/kg and 60 mg/kg, respectively [130], treatment with another PDE4 inhibitor, rolipram, has appeared to exacerbate periventricular WMHs in rat pups [131]. Despite not affecting WMH progression in stroke- and dementia-free subjects, the PDE3 inhibitor cilostazol has demonstrated an acceptable safety profile [132]. In a small-sized clinical study, tadalafil, a PDE5 inhibitor, has been shown to improve perfusion in the cerebral microvasculature in patients with cerebral small vessel disease stroke. The increased oxygenation positions tadalafil as an attractive new therapeutic target in cSVD [133]. However, well-designed future studies are needed to evaluate the effects of long-term PDE inhibitor treatment on cerebrovascular reactivity, endothelial function, and general neurovascular changes, including WMHs in cSVD and stroke.

### 4.5. Oral Antihyperglycaemic Drugs

Diabetes mellitus (DM), characterised by chronic hyperglycaemia, causes oxidative stress, vascular inflammation, and endothelial dysfunction and constitutes one of the main risk factors for cSVD [134]. Oral forms of sodium–glucose cotransporter-2 (SGLT2) inhibitors and glucagon-like peptide-1 receptor agonists (GLP-1 RAs) represent the commonly prescribed medications to control this condition [135,136]. High levels of blood glucose impair endothelial NO production, reduce vascular reactivity, increase permeability, and potentiate the production of several pro-inflammatory cytokines such as TNF-α and IL-6 [45]. Protein glycation and the formation of advanced glycation end products (AGEs) play a critical role in the development of various hyperglycaemia-induced medical complications like retinopathy, cardiomyopathy, and neuropathy. Increased production of ROS, mediated in a large part by the activation of PKC and NADPH oxidase, are also known to contribute to neurovascular degeneration [137,138,139,140]. Impaired insulin signalling and associated insulin resistance in diabetics further exacerbate cerebral and neuronal damage [134].

Metformin, a commonly prescribed biguanide, has an effect by activating the AMP-activated protein kinase. Activation of this enzyme leads to decreased expression of gluconeogenic enzymes like phosphoenolpyruvate carboxykinase and glucose-6-phosphatase, preventing hepatic glucose output, and augments insulin sensitivity in peripheral tissues [141]. Therefore, it aids in restoring endothelial function by lowering the levels of ROS and inflammatory substances. These concurrent reductions in oxidative stress and inflammation enhance cerebral perfusion and abate WMHs [142]. Similar to GLP-1 RAs, SGLT2 inhibitors also exert their beneficial effects on neurovasculature by reducing oxidative stress and inflammation. Reduced neuroinflammation in turn leads to diminished WMH levels and BBB permeability [135,143]. Another group of oral antidiabetic agents, namely dipeptidyl peptidase-4 (DPP-4) inhibitors, also elevate insulin sensitivity by increasing endogenous GLP-1 levels. As a result, they reduce inflammation and improve endothelial function [144].

### 4.6. Tetracycline Antibiotics

Beyond their antimicrobial effects, tetracycline antibiotics show anti-inflammatory properties and can modulate MMP activity in the host organism. By chelating the zinc and calcium ions, essential minerals for MMP function, tetracyclines inhibit MMP activity [145]. Due to this inhibitory effect on MMPs and their modulatory effects on mitochondrial function, tetracyclins exhibit anti-inflammatory properties and help successfully reduce chronic neuroinflammation by suppressing pro-inflammatory mediators such as TNF-α and IL-6 [146]. By inhibiting MMPs, tetracyclines prevent the degradation of extracellular matrix components and TJ proteins and thus help preserve BBB integrity [147,148]. Tetracyclines tend to accumulate in host mitochondria owing to their similarity to bacterial ribosomes and mitochondrial ribosomes. They can affect mitochondrial respiration and ATP production by inhibiting mitochondrial protein synthesis. As a result, they reduce mitochondrial ROS production, prevent oxidative damage, and attenuate microglial activation [149,150]. Additionally, they contribute to the preservation of white matter integrity by regulating apoptotic pathways [151].

### 4.7. Anti-Anginal Drugs

cSVD and coronary artery disease share many aetiological similarities, including endothelial dysfunction, impaired autoregulation, and ischaemia. Hence, pharmacological interventions used to treat angina are expected to produce positive responses in case of cSVD [152,153]. Pharmacological agents utilised for the treatment of angina pectoris encompass a variety of drug classes, including nitrates, calcium channel blockers, and β-adrenergic antagonists, as well as specific medications like ranolazine, nicorandil, and ivabradine.

#### 4.7.1. Nitrates

NO donors, such as nitrates, enhance vasodilation and reduce vascular resistance. In smooth muscle, NO activates soluble guanylyl cyclase, stimulating the production of cGMP. Increased levels of cGMP activate cGMP-dependent protein kinase (PKG). cGMP and PKG reduce the Ca^2+^ sensitivity of smooth muscle. Ca^2+^ causes a decrease in the activity of calmodulin-activated myosin light-chain kinase and muscle relaxation [154,155]. Thus, they regulate CBF and mitigate ischaemic damage [156]. Additionally, nitrates may have a protective effect by reducing the excessive Ca^2+^ influx seen in nerve damage [157].

#### 4.7.2. Calcium Channel Blockers

Calcium channel blockers (CCBs) reduce the influx of Ca^2+^ into vascular smooth muscle, decrease vascular tone, and, consequently, increase blood flow [158]. By preventing the entry of extracellular Ca^2+^ into cells through voltage-gated Ca^2+^ channels, CCBs markedly decrease peripheral vascular resistance and blood pressure. While CCBs like nifedipine and felodipine exert vasodilator effects by inhibiting high-voltage-activated L-type channels, those such as amiodipine and clinidipine block high-voltage-activated N-type channels and reduce the release of norepinephrine, a vasoconstrictor, from sympathetic nerve endings. CCBs such as efonidipine and azeinidipine, on the other hand, inhibit low-voltage-activated channels. It is therefore thought that CCBs that inhibit L- and N-type channels may have organ-protective effects [159]. By blocking the entry of basal Ca^2+^ into cerebral arteries, they can increase CBF and protect the neurovascular system against various cerebral degenerative diseases, such as subarachnoid haemorrhage, known to be associated with excessive availability of intracellular Ca^2+^ [158].

#### 4.7.3. β-Adrenergic Receptor Antagonists

The β-adrenergic receptor antagonists (β-blockers) are a family of agents that are used to treat hypertension and cardiac arrhythmias. The β-blockers compete with β-adrenergic agonists, e.g., epinephrine and norepinephrine, to bind beta-receptor sites. They are often divided into “selective” and “non-selective” agents based on their ability to block β-1 receptors that are found in cardiac muscle and β-2 receptors found in bronchial and smooth muscles. While β-1 selective blockers are used to treat heart disease, the nonselective β-blockers are predominantly used to control recurrent haemorrhages observed in patients with cirrhosis and portal hypertension [160,161].

Blockade of β1-adrenergic receptors aids vascular relaxation by preventing hyperpolarization of intermediate-conductance Ca^2+^-activated potassium channels (IKCa) in vascular ECs [162]. Another study investigating the effects of selective and non-selective α-1, β-1, α-2, and β-2 blockers on regional CBF responses to hyperbaric hyperoxia concluded that only propranolol, a non-selective β-1 and β-2 blocker, regulated CBF [163].

#### 4.7.4. Others

Ranolazine, an agent used to treat chronic angina, has been shown to mitigate myocardial infarction by suppressing late sodium ion channel activity, which leads reduced intracellular and mitochondrial Ca^2+^. As a result, it prevents cell damage by inhibiting the suppression of the ROS-scavenging feature of mitochondria. This property suggests that it may enhance mitochondrial function and attenuate neuroinflammation in cerebral ischaemia [164]. Ivabradine, a hyperpolarization-activated cyclic nucleotide-gated channel blocker, specifically suppresses the electrical current in the sinoatrial node to slow the heart rate, whereby it enhances diastolic perfusion and, as a consequence, improves cerebral perfusion [165]. Nicorandil, a vasodilator used to treat angina, functions as both a nitrate and a potassium channel activator. Its nitrate characteristic facilitates vasodilation by increasing NO levels, while its potassium channel opening action enhances smooth muscle relaxation through cellular hyperpolarization [166].

## 5. cSVD and Clinical Trials

The findings of the clinical trials conducted with the drugs mentioned in this review and the cSVD keywords are summarised in Table 1. Clinical studies mostly investigate the efficacy of drugs. However, a few of them also investigate the basic pathologies of the disease. The number of studies on cSVD appears to have risen significantly since 2020.

## 6. Discussion and Conclusions

Cardiovascular diseases remain the leading cause of death globally, with dementia now following closely. Due to an increase in life expectancy, dementia has actually become the primary cause of death in some countries. Many risk factors such as hypertension, hyperglycaemia, smoking, and sleep apnoea contribute to the rise of both cardiovascular diseases and dementia, which appear to share similar changes in vascular structure and function. Lacunar stroke is one of the subtypes of ischemic stroke. It is characterised by the appearance of lacunar infarcts, which may represent the first recognisable sign of cSVD, affecting the small perforating arteries, capillaries, and venules of the brain. Owing to the spatial resolution issues in neurovascular imaging, infrequency of pathological studies, and lack of appropriate experimental model availability, the aetiology of a perforating artery occlusion remains unclear [174]. Even so, the pathological changes observed in cSVD are known to include oxidative stress, inflammation, ischaemia due to nitric oxide deficiency, loss of tight junction proteins in ECs caused by MMP activation, and, ultimately, endothelial dysfunction, an early change in cSVD. Endothelial dysfunction primarily disrupts the BBB permeability and accelerates the leakage of toxic components from the blood to the brain parenchyma. This triggers neuroinflammation and neurodegeneration and, as a consequence, promotes neurological damage and dementia. Scrutiny of the therapeutic options that can preserve BBB integrity and normal endothelial function is of crucial importance for patients diagnosed with cSVD.

Advancements in drug repositioning studies and emerging technologies have led to a better understanding of the mechanisms underlying both diseases and the available drugs. This review discusses medications currently used to treat dementia and neurological disorders, as well as those preferred for managing vascular diseases and hyperglycaemia, which is closely associated with cSVD. As summarised in Table 2, these compounds have been found to promote vasodilation by increasing NO levels through various pathways, and to decrease levels of ROS and pro-inflammatory cytokines. Tetracycline antibiotics and lithium drugs have also been observed to preserve BBB integrity by inhibiting MMPs. Investigating the direct relationship between these drugs and cSVD is necessary. Determining their effects on endothelial function and the NVU presents a valuable first step in this regard. In vitro BBB models are among the most valuable tools for investigating diseases like cSVD, whose underlying molecular mechanisms remain incompletely elucidated, as well as for examining the effects of pharmacological interventions. Transwell-based triple or quadruple co-culture systems derived from human primary cells represent the optimal models in this context, as they are time-efficient, cost-effective, and enable detailed investigation of the molecular pathways involved. The data obtained from these studies will provide a foundation for a healthier and more ethically grounded transition to translational and clinical research. In conclusion, there is a need to improve our understanding of the molecular pathologies underlying cSVD and explore potential pharmacological interventions through well-designed preclinical studies. The data generated will help inform the design of future clinical studies. For an update on lacunar stroke pathophysiology and a clinical perspective on managing lacunar strokes, readers are advised to read recent reviews discussing the relevant imaging and translational studies [174].

## Figures and Tables

**Table 1:** Pharmacological interventions with agents targeting cSVD or associated cognitive impairments.

Clinical Trial ID	Study Focus	Findings	Ref.
ChiCTR-IOR-17013557/ChiCTR-EOC-017013598	Statin therapy in adults ≥ 75 years with cSVD	Statin therapy alleviated the progression of WMH, lacunes, and EPS without increasing the risk of CMHs, suggesting it as an efficient and safe intervention for cSVD in older adults.	[167]
NCT00103948	Efficacy, safety, and tolerability of donepezil HCl in patients with CADASIL who have cognitive impairment	While no change was observed in the vascular AD assessment scale cognitive subscale, an improvement was observed in the Trail Making Test Part B.	[168]
NCT02444637	Effectiveness of Rivastigmine in subjects with AD and cerebrovascular disease	No results posted	^1^
NCT00163150	Evaluation of the efficacy of atorvastatin treatment for 3 months (80 mg/day) on cerebral vasoreactivity in lacunar patients	There was no positive effect of 3-month treatment with atorvastatin on severe cerebral microvasculature endothelial dysfunction in patients with lacunar stroke.	[169]
NCT03855332	Examines the effects of sildenafil, a PDE5 inhibitor, on cerebrovascular pulsatility and reactivity, and compares its efficacy to that of cilostazol, a PDE3 inhibitor	Lowering cerebral pulsatility and improving cerebrovascular reactivity with sildenafil treatment may indicate potential benefits in preventing small vessel disease progression.	[170]
NCT06175663	Studies abnormalities in brain structure and vascular function in young hypertensive patients. Also determines the effects of blood pressure changes, during a period of withdrawal and restart of blood pressure medications, on brain structure and vascular function	No results posted	^1^
NCT05169021	Evaluates the impact of intensive antihypertensive therapy versus standard antihypertensive therapy in reducing the risk of combined cardiovascular and cerebrovascular events in cSVD patients with hypertension and elevated homocysteine levels, using two common antihypertensive drugs: amlodipine 5 mg tablets or amlodipine–folic acid 5.8 mg tablets	No results posted	^1^
NCT03451591	Investigates the feasibility, safety, and effects of a 1-year treatment using ISMN and cilostazol on blood vessel health, daily functioning, and cognitive abilities in patients who have had a lacunar stroke	The LACI-2 trial was successful, and ISMN and cilostazol were safe and well-tolerated. These medications may help prevent additional strokes, disability, and cognitive issues after lacunar stroke. They can also avoid other negative outcomes in cerebral small vessel disease.	[171]
NCT06715007	Evaluates the effect of different antiplatelet agents (e.g., cilostazol) on cSVD and the retina in patients with cSVD (recent small subcortical infarcts or WMH, respectively)	No results posted	^1^
NCT04753970	Tests whether impaired cerebral and retinal vasoreactivity may serve as biomarkers for SVD progression, and evaluates the safety and efficacy of cilostazol (antiplatelet agent with vasodilatory and anti-inflammatory properties) for the treatment of SVD	No results posted	^1^
NCT02481323	Investigates if short-term ISMN and cilostazol, alone or in combination, improves magnetic resonance imaging-measured cerebrovascular function in patients with lacunar ischemic stroke	It is demonstrated that measuring cerebrovascular function with magnetic resonance imaging is feasible in clinical trials and that ISMN and cilostazol may improve cerebrovascular function.	[172]
NCT01932203	Assesses if there is difference in the efficacy of cilostazol and aspirin in impacting the progression of white matter changes in cSVD	No results posted	^1^
NCT00741286	Investigates if cilostazol decrease the vascular resistance in patients with acute lacunar infarction and decreases the PIs in patients with acute lacunar infarction	Cilostazol reduced transcranial Doppler PIs more than placebo at 90 days in acute lacunar infarction. This may be due to its additional effects, like vasodilation, beyond just its antiplatelet action.	[173]
NCT06649240	Investigates the optimal LDL-cholesterol target for patients with small vessel occlusion stroke	No results posted	^1^
NCT06031610	Determines if carotid artery stenosis revascularization, compared to standard medication treatment alone, can effectively reduce the progression of cSVD burden and improve the severity of retinal issues and cognitive impairment	No results posted	^1^
NCT05356104	Investigates the safety and efficacy of the GLP-1R agonist in patients with moderate-to-severe cSVD	No results posted	^1^
NCT05710367	Tests dapagliflizone in Fabry patients for kidney and heart function improvement	No results posted	^1^

^1^ Relevant clinical trial information can be found at www.clinicaltrials.gov (accessed on 15 February 2025) via the clinical trial ID. cSVD: cerebral small vessel disease, CMHs: Cerebral microhaemorrhages, WMH: White matter hyperintensity, AD: Alzheimer’s disease, ISMN: Isosorbide mononitrate, EPS: Enlarged perivascular space, PDE: Phosphodiesterase, PI: Pulsatility index, LDL: Low-density lipoprotein, LACI-2: Lacunar Intervention Trial-2, GLP-1R: Glucagon-like peptide-1 receptor.

**Table 2:** The effects of pharmacological agents included in the review on cerebral small vessel disease (cSVD).

Drug Category	Major Effects
Acetylcholine Esterase Inhibitors	Enhance ACh levels in synaptic cleft. Improve cholinergic signalling. Reduce neuroinflammation (via cholinergic anti-inflammatory pathway). Improve BBB integrity and reduce oxidative stress. Examples: Donepezil, Rivastigmine, Galantamine.
HMG-CoA Reductase Inhibitors (Statins)	Lower LDL cholesterol levels. Anti-inflammatory and anti-proliferative effects. Improve endothelial function and NO bioavailability. Reduce oxidative stress and enhance BBB integrity. Examples: Atorvastatin, Simvastatin.
Lithium Drugs	Regulate neural signalling via GSK-3 inhibition. Promote neuroplasticity and neurogenesis. Reduce ROS production and oxidative stress. Enhance mitochondrial function and BBB integrity. Examples: Lithium Carbonate, Lithium Citrate.
Phosphodiesterase Inhibitors	Increase cAMP/cGMP levels. Promote NO-mediated vasodilation. Reduce inflammation by lowering TNF-α and IL-6. Preserve BBB integrity. Examples: PDE3 (Cilostazol), PDE4 (Roflumilast), PDE5 (Sildenafil).
Oral Antihyperglycaemic Drugs	Reduce oxidative stress and vascular inflammation. Improve endothelial function and insulin sensitivity. Examples: Metformin (AMPK activator), SGLT2 inhibitors, GLP-1 receptor agonists, DPP-4 inhibitors.
Tetracycline Antibiotics	Anti-microbial. Anti-inflammatory via MMP inhibition. Reduce ROS and preserve BBB integrity. Prevent microglial activation and oxidative damage. Examples: Doxycycline, Minocycline.
Anti-Anginal Drugs	Enhance vasodilation via NO and cGMP pathways. Improve cerebral perfusion and reduce ischaemic damage. Examples: Nitrates (Nitroglycerin), Calcium Channel Blockers, β-blockers, Ranolazine, Ivabradine, Nicorandil.

ACh: Acetylcholine, LDL: Low-density lipoprotein, NO: Nitric oxide, BBB: Blood–brain barrier, GSK-3: Glycogen synthase kinase-3, ROS: Reactive oxygen species, cAMP: Cyclic adenosine monophosphate, cGMP: Cyclic guanosine monophosphate, PDE: Phosphodiesterase, AMPK: AMP-activated protein kinase, SGLT2: Sodium–glucose cotransporter-2, GLP-1: Glucagon-like peptide-1, DPP-4: Dipeptidyl peptidase-4.
